# Supplementary material for: Fatty acid synthase mediates EGFR palmitoylation in EGFR mutated non‐small cell lung cancer
Source: EMBO Mol Med. 2018 Feb 15;10(3):e8313. doi: 10.15252/emmm.201708313 (PMC5840543; doi:10.15252/emmm.201708313)
Supplement: Supplementary file 1 — Appendix [file EMMM-10-e8313-s001.pdf]

Fatty Acid Synthase mediates EGFR palmitoylation in EGFR mutated Non Small Cell Lung Cancer

Azhar Ali, Elena Levantini, Teo Jun Ting, Julian Goggi, John G. Clohessy, Wu Chan Shuo, Polly Chen Leilei, Henry Yang, Indira Krishnan, Olivier Kocher, Zhang Junyan, Ross Soo, Kishore Bhakoo, Chin Tan Min, Daniel G Tenen

Appendix

Table of contents **Page**

Table of contents	Page
Appendix Fig S1 with legend	1
Appendix Fig S2 with legend	2
Appendix Fig S3 with legend	3-4
Appendix Fig S4 with legend	5-6
Appendix Fig S5 with legend	7
Appendix Fig S6 with legend	8-9
Appendix Fig S7 with legend	10
Appendix Fig S8 with legend	11
Appendix Fig S9 with legend	12-13
Appendix Fig S10 with legend	14
Appendix Fig S11 with legend	15
Appendix Fig S12 with legend	16
Appendix Fig S13 with legend	17
Appendix Fig S14 with legend	18
Appendix Fig S15 with legend	19
Appendix Fig S16 with legend	20
Appendix Fig S17 with legend	21
Appendix Fig S18 with legend	22
Appendix Fig S19 with legend	23-29
Appendix Table S1 with legend	30
Appendix Table S2 with legend	31

Appendix Table S3 with legend 32

Appendix Table S4 with legend 33

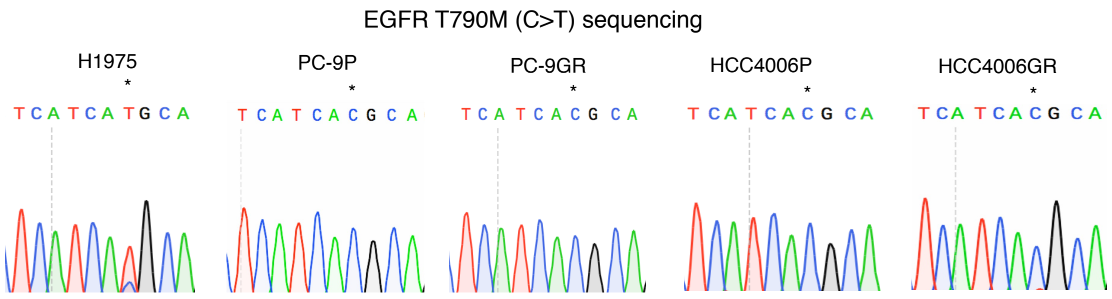

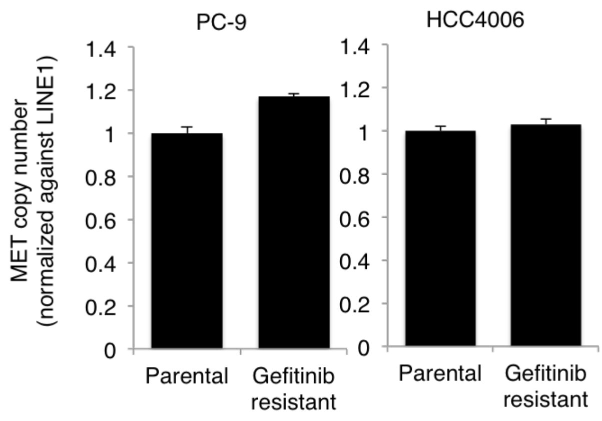

Appendix Fig S1. DNA analyses of PC-9 and HCC4006 cells to determine EGFR T790M mutation and MET amplification status. H1975 acts as positive control for EGFR T790M sequencing.

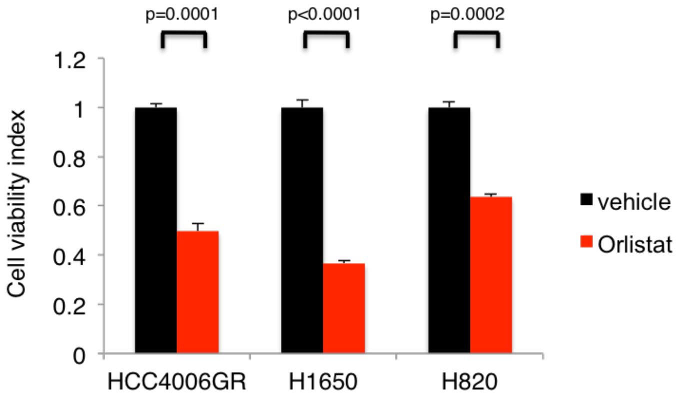

Appendix Fig S2. Cell viability assays showing the effects of Orlistat on HCC4006GR, H1650 and H820 EGFR mutated NSCLC cells with TKI resistance. Cells were exposed to 100 μM of Orlistat for 72 h. Significance in differences in viability indexes, in which vehicle acted as control, was determined by t test.

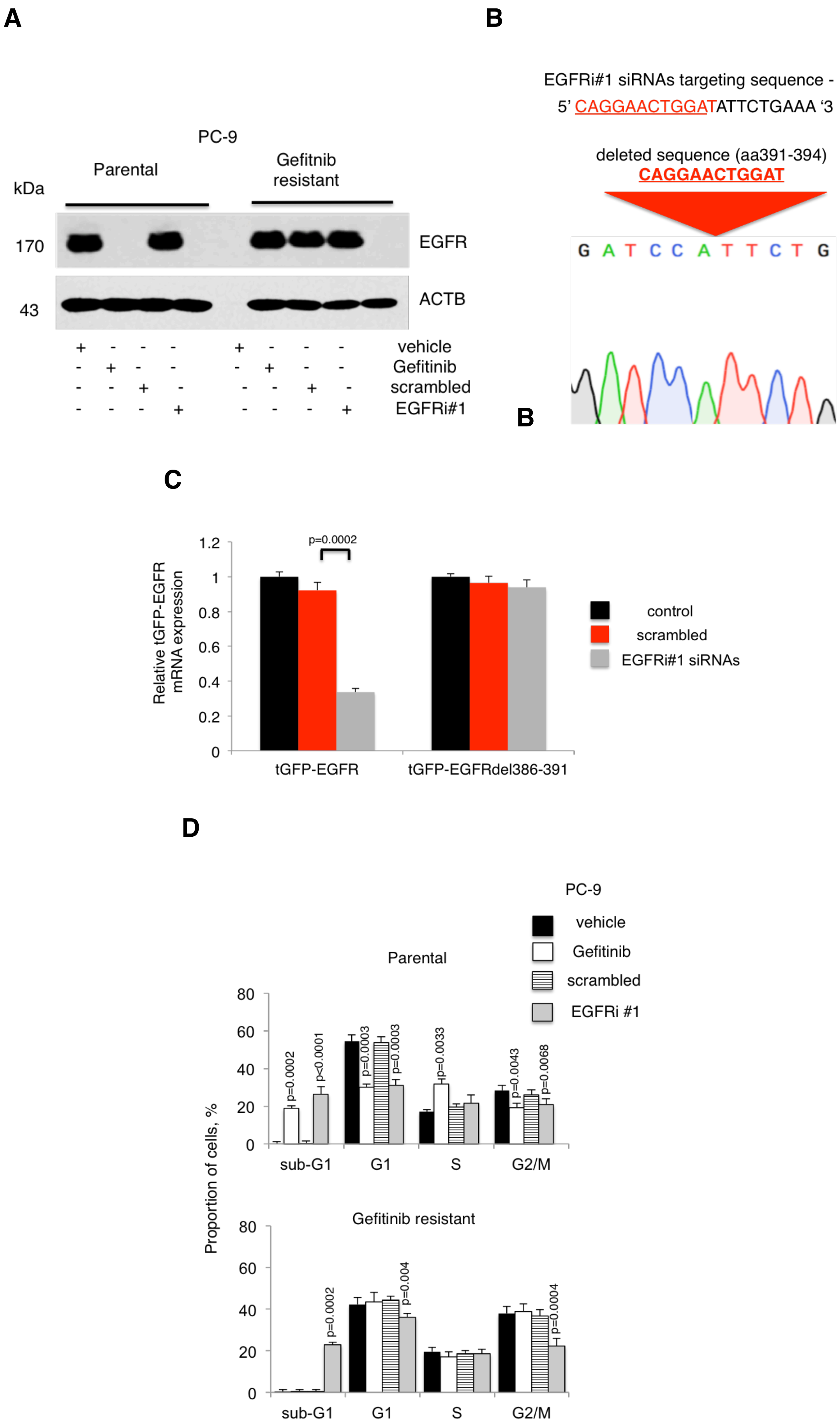
A
B
EGFRi#1 siRNAs targeting sequence -
5' CAGGAACTGGATATTCTGAAA '3
PC-9
Parental
Gefitinib resistant
kDa
170
43
EGFR
ACTB
vehicle
Gefitinib
scrambled
EGFRi#1
deleted sequence (aa391-394)
CAGGAACTGGAT
G A T C C A T T C T G
B
C
p=0.0002
Relative tGFP-EGFR mRNA expression
1.2
1
0.8
0.6
0.4
0.2
0
tGFP-EGFR
tGFP-EGFRdel386-391
control
scrambled
EGFRi#1 siRNAs
D
PC-9
vehicle
Gefitinib
scrambled
EGFRi #1
Parental
Proportion of cells, %
80
60
40
20
0
p=0.0002
p<0.0001
p=0.0003
p=0.0003
p=0.0033
p=0.0043
p=0.0068
sub-G1
G1
S
G2/M
Gefitinib resistant
p=0.0002
p=0.004
p=0.0004

Appendix Fig S3. Generation of EGFRi#1 resistant EGFR cDNA to validate EGFRi#1 siRNAs specificity.

A. Western blot data showing parental and Gefitinib resistant PC-9 cells exposed to gefitinib (50 nM), and 25 nM of scrambled siRNAs, or EGFR siRNAs for 72 h. ACTB was selected as a housekeeping gene control.

B. EGFRi#1 resistant tGFP-tagged EGFR cDNA (EGFRdel386-391) was generated by deleting 12 of 21 nucleotides of the EGFR sequence (underlined in red) targeted by EGFRi#1 siRNAs by site-directed mutagenesis. The success of mutagenesis was verified by sequencing, as shown in the sequencing chromatogram.

C. Quantitaive PCR showing that tGFP-tagged EGFRdel386-391 was resistant/untargetable to EGFRi#1 siRNAs, when compared to tGFP-tagged EGFR. NL20 cells were co-transfected with tGFP-tagged EGFR or tGFP-tagged EGFRdel386-391, and EGFRi#1 siRNAs for 72 h. This was followed by cell harvesting, RNA isolation and the measurement of tGFP-tagged EGFR mRNAs using tGFP-specific primers (**Table S4**). Significance in differences in mRNA expression, in which scrambled acted as control, was determined by t test.

D. Cell cycle analysis of parental and gefitinib resistant PC-9 cells exposed to either scrambled siRNAs or Gefitinib or EGFR siRNAs for 72 h. Significance in differences in percentages of cells, in which scrambled acted as control, was determined by t test.

A

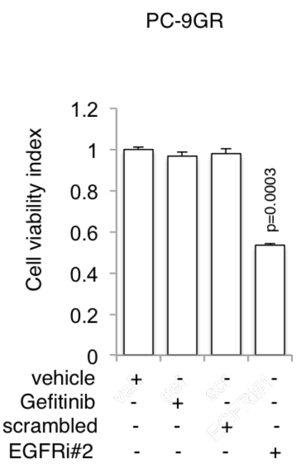

B

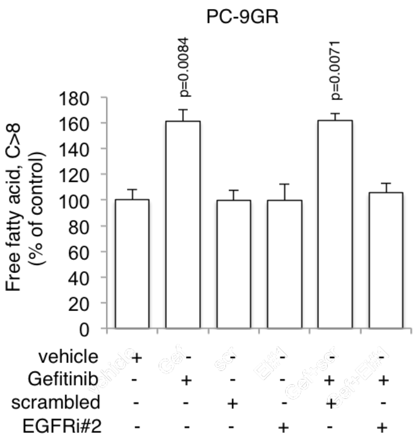

C

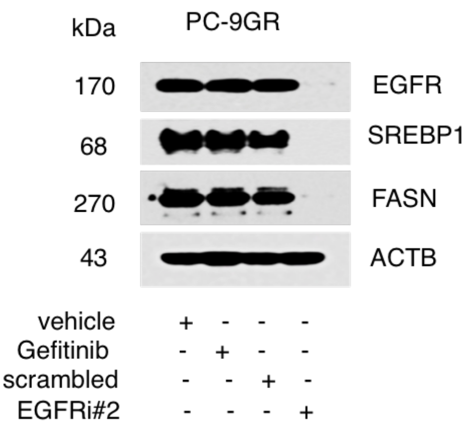

Appendix Fig S4. EGFR knockdown with a second EGFR siRNAs (EGFRi#2) in PC-9GR cells.

A. Cell viability assays on cells exposed to Gefitinib or EGFR siRNAs for 72 h. Significance in differences in cell viability indices, in which scrambled acted as control, was determined by t test.

B. Measurement of cellular free fatty acid after Gefitinib treatment or EGFR knockdown for 72 h. Significance in differences in FFAs levels, in which vehicle acted as control, was determined by t test.

C. Western blot analysis of cells treated with either vehicle, Gefitinib, scrambled or EGFR siRNAs for 72 h. ACTB was selected as a housekeeping gene control. Vehicle or scramble treated cells acted as controls.

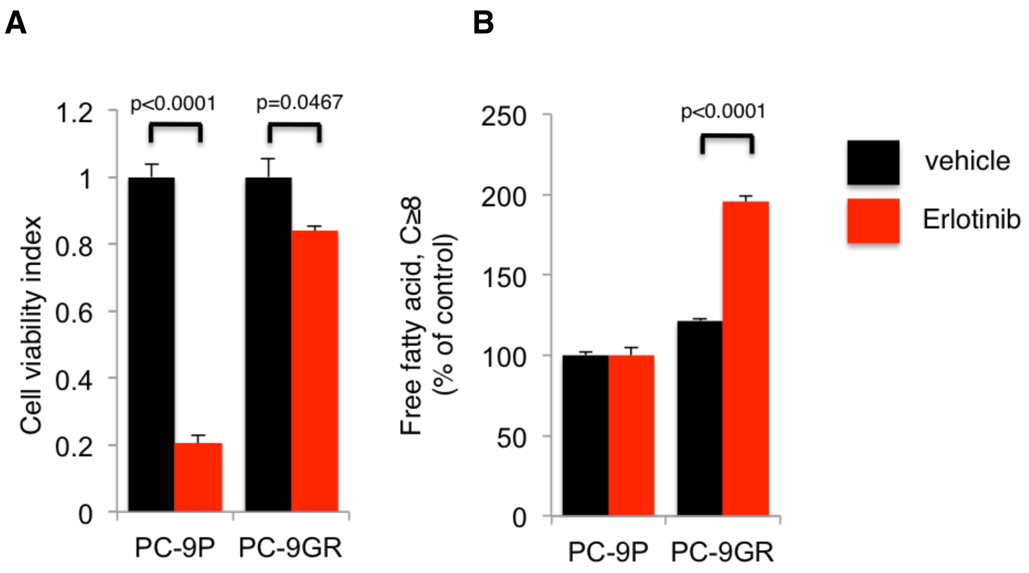

Appendix Fig S5. Cell viability and cellular FFAs measurement in PC-9P and PC-9GR cells treated with Erlotinib at 1 μM for 72 h.

A. Cell viability assay.

B. Cellular FFAs assay.

Significance in differences in viability and FFAs levels, in which vehicle acted as control, was determined by t test.

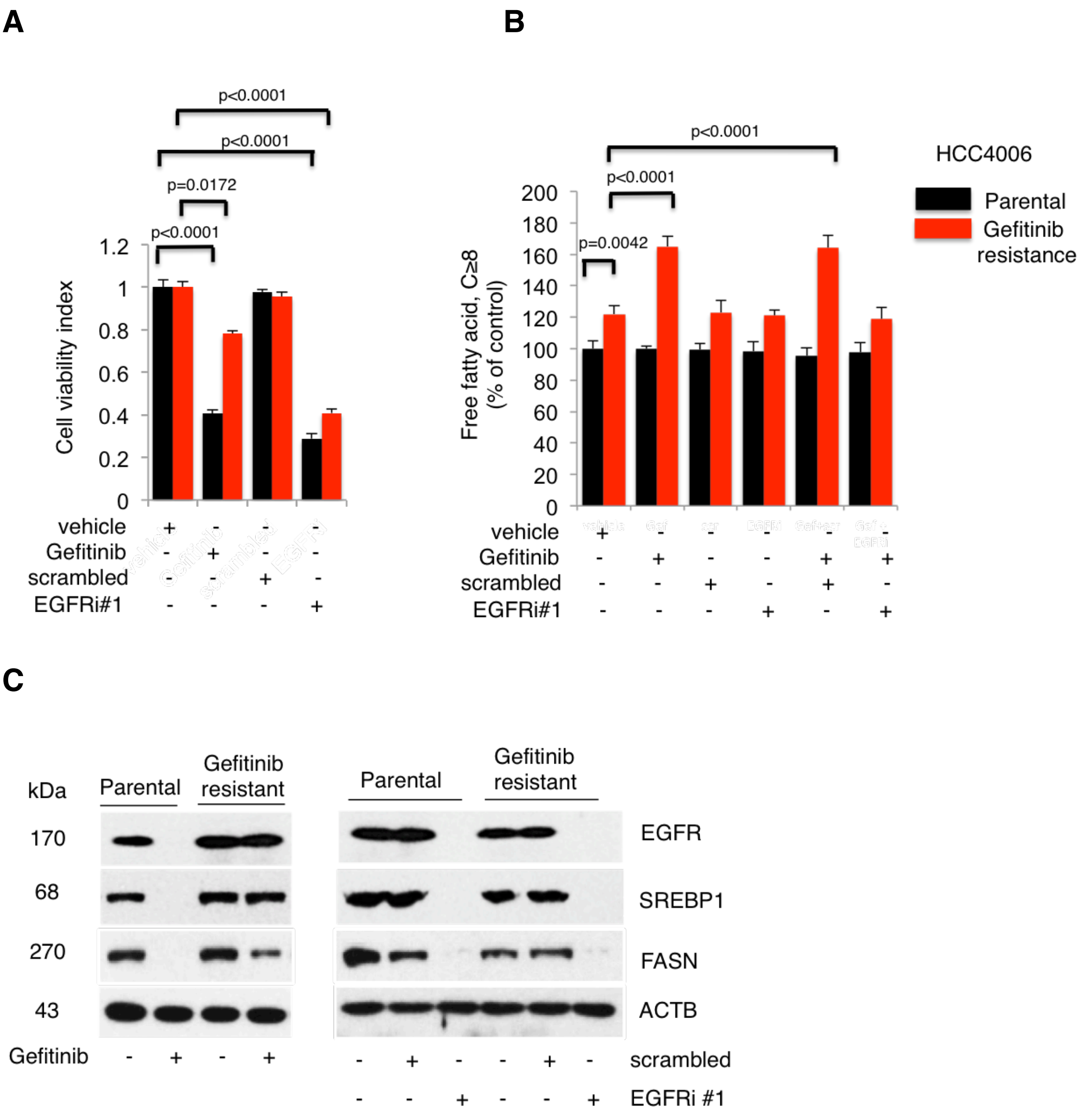

Appendix Fig S6. The EGFR-FASN signaling network is active in TKI resistant HCC4006GR NSCLC cells.

A. Cell viability assays to determine the effects of Gefitinib (1 μM) or EGFR knockdown (EGFRi#1 at 25 nM) exposure on EGFR del746-749/A750P HCC4006GR cells after 72 h. Significance in differences in viability indexes, in which vehicle acted as control, was determined by t test.

B. Measurement of cellular free fatty acid (FFA) after Gefitinib or EGFR knockdown for 72 h. Significance in differences in cellular FFAs, in which vehicle acted as control,

was determined by t test. **C.** Western blot analysis of HCC4006P and HCC4006GR cells. Cells were treated with vehicle (-) or Gefitinib (+) for 72 h (left panel). Cells were exposed to either scrambled siRNA or EGFR siRNAs for 72 h (right panel). Vehicle or scrambled treated cells acted as controls. ACTB was selected as a housekeeping gene control.

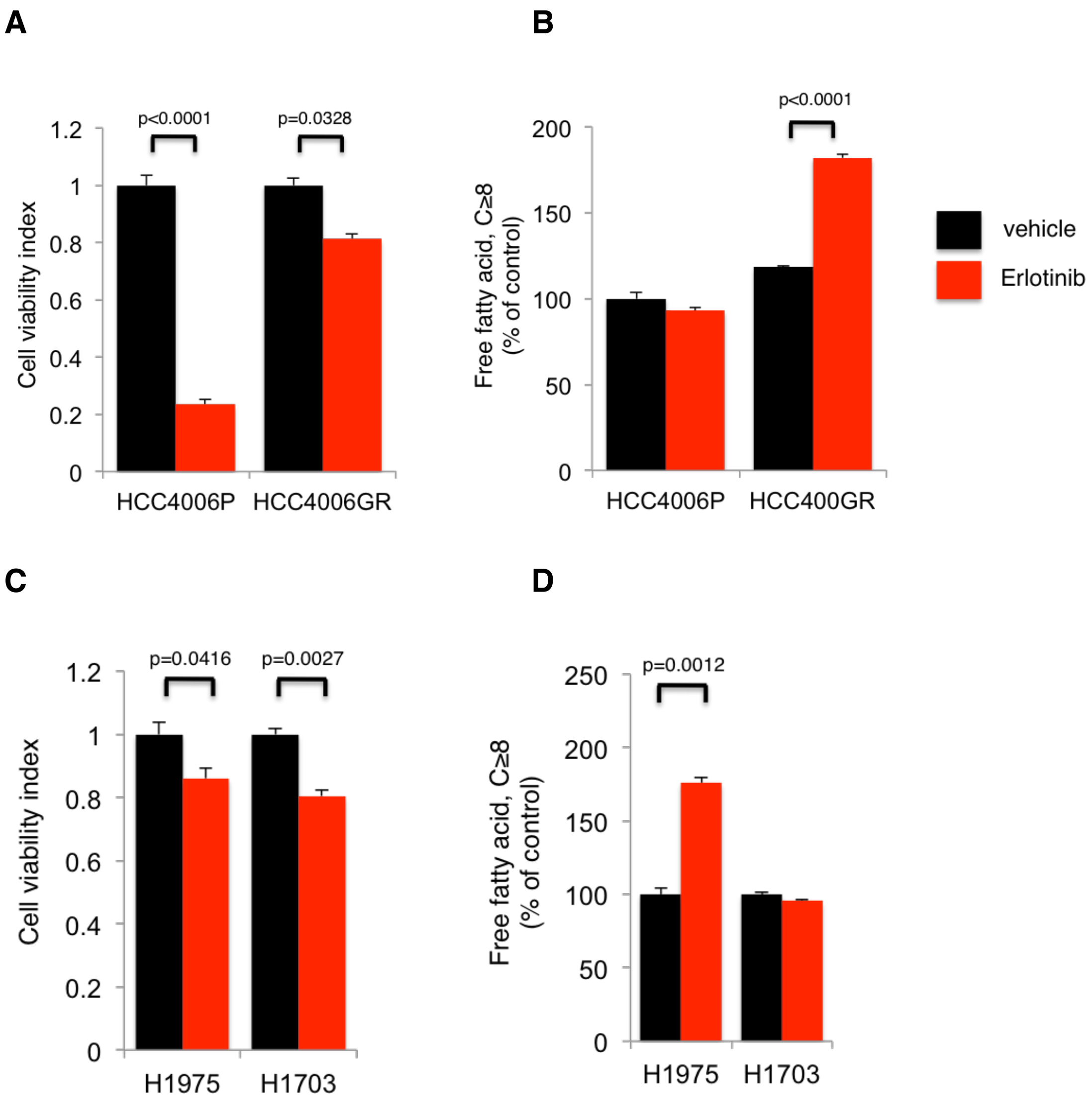

Appendix Fig S7. The effects of Erlotinib (at 1 μM for 72 h) on cellular viability (**A and C**) and FFAs (**B and D**) in isogenic HCC4006, H1975 and H1703 NSCLC cells. Significance in differences in cellular viability and FFAs levels, in which vehicle acted as control, was determined by t test.

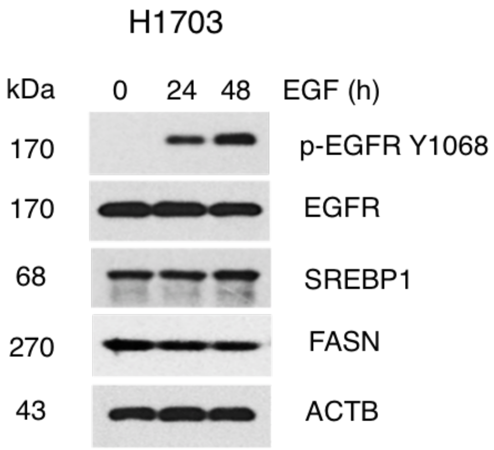

Appendix Fig S8. Western blot images showing phospho-EGFR (Y1068), total EGFR, SREBP1 and FASN expression after EGF (10 ng/ml) were added to starving H1703 cells at 0, 24 and 48 h. ACTB was selected as a housekeeping gene control.

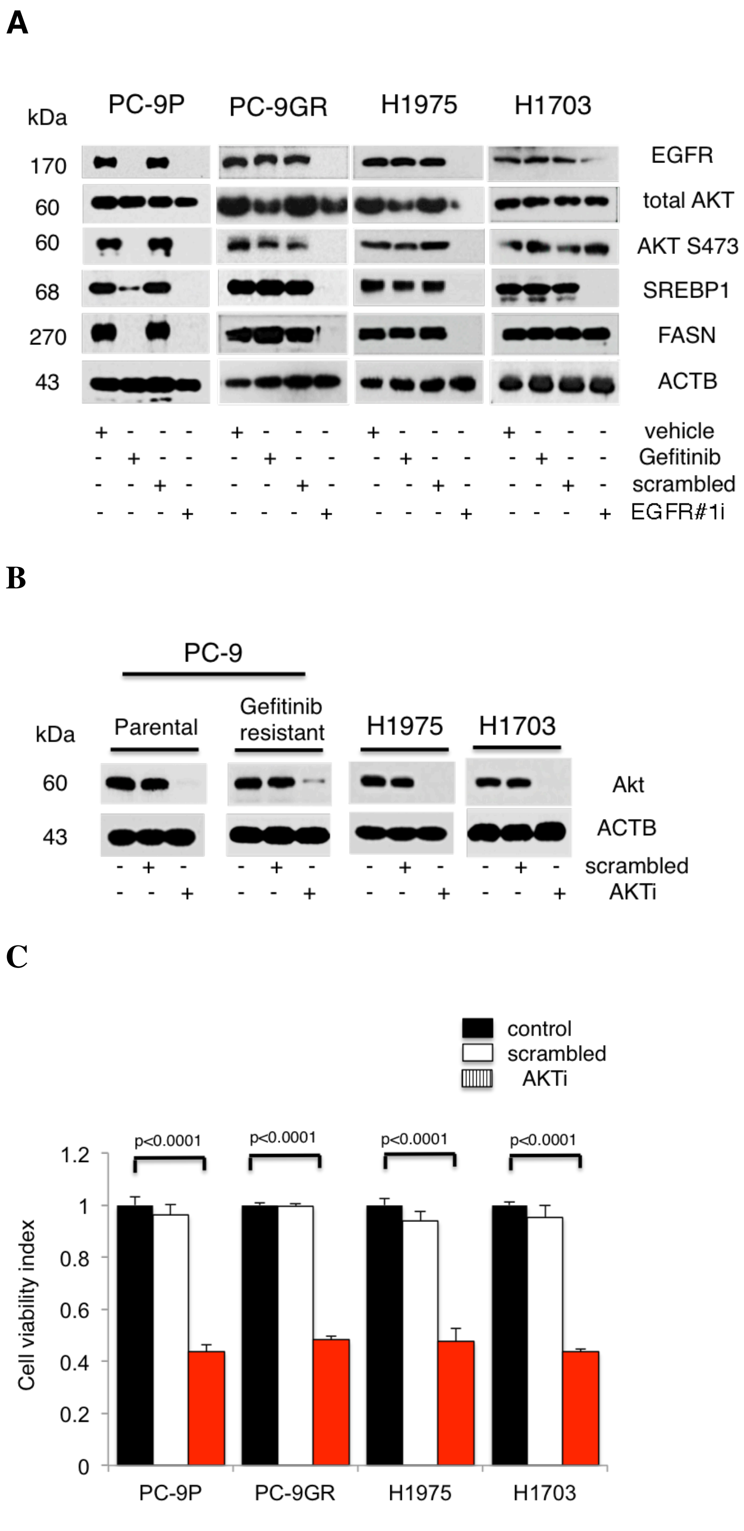

Appendix Fig S9. TKI resistance is associated with active Akt signaling in EGFR mutant cells.

A. Western blot analysis of protein lysates from cells exposed to either Gefitinib (50 nM for PC-9P; 1 μM for PC-9GR, H1975, and H1703) or EGFR knockdown for 72 h.

Membranes were probed with antibodies against the indicated proteins. ACTB was selected as a housekeeping gene. Vehicle or scrambled treated cells acted as control.

B. Cells were exposed to scrambled or Akt siRNAs (AKTi) for 72 h followed by cell viability assays. Significance in differences in cellular viability, in which Scrambled-treated cells acted as control, was determined by t test.

C. NSCLC cells were treated with scrambled or Akt siRNAs for 72 h prior harvesting and subsequent Western blotting. ACTB was selected as a housekeeping gene control.

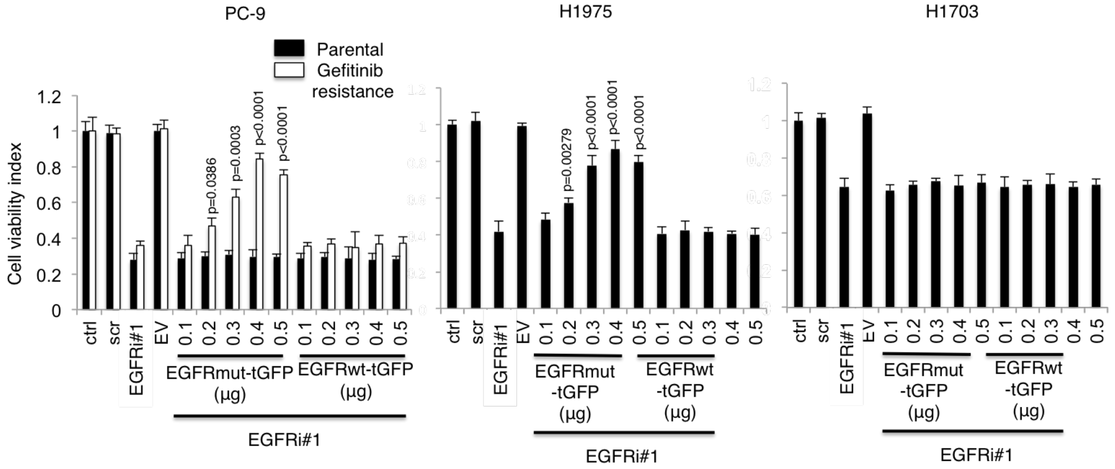

Appendix Fig S10. Cell viability assays of EGFR knockdown NSCLC cells transfected with either GFP-tagged EGFR wild type, ΔE746-A750, or L858R/T790M constructs. Cells were allowed to attach overnight in 96-well format prior to transfection with EGFR siRNAs (EGFRi#1) for 12 h. This was followed by transfection with EGFR constructs ranging from 0.1 to 0.5 μg in increments of 0.1 μg for additional 60 h (for a total of 72 h). Significance in differences in cellular viability, in which EGFRi#1-treated cells acted as control, was determined by t test.

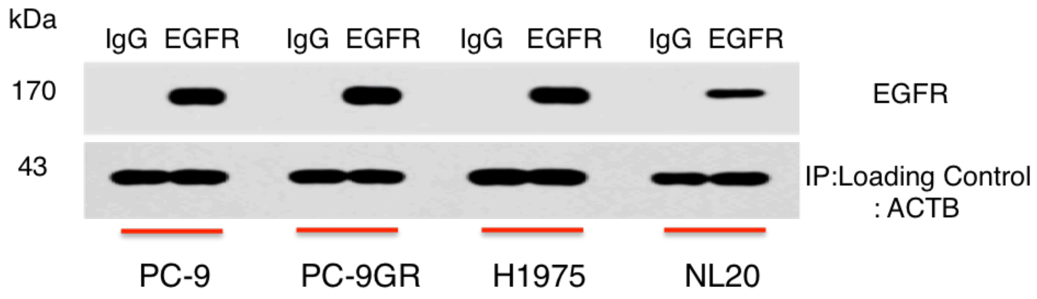

Appendix Fig S11. Western blotting showing immunoprecipitation of EGFR from NSCLC and immortalized nontransformed lung cells.

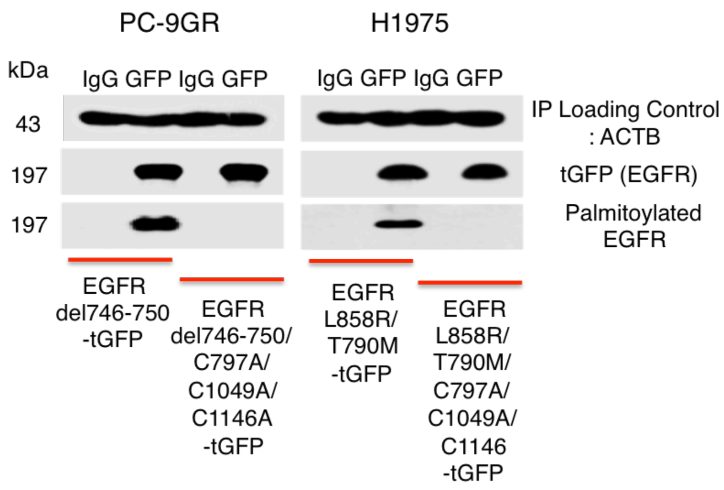

Appendix Fig S12. Western blot images showed palmitoylation status of EGFR in palmitoylation-deficient tGFP-tagged EGFR mutant (del746-750 and L858R/T790M) constructs that were mutated at cysteine residues 797, 1049 and 1146 to alanine.

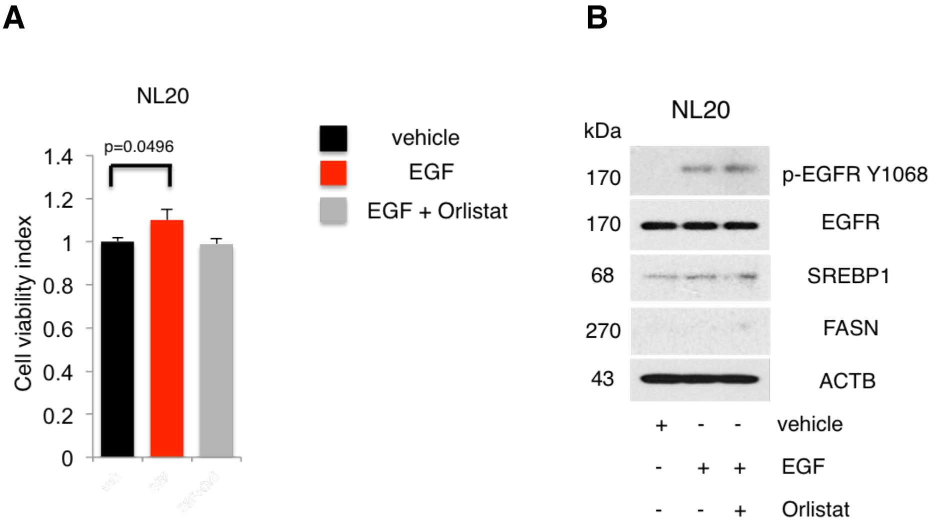

Appendix Fig S13. The effects of EGFR stimulation by EGF on NL20 cells. Cells were grown in reduced serum (1% FBS) for 48 h prior to the experiment.

A. Cell viability assays showing the NL20 cells exposed to vehicle, EGF (10 ng/ml) alone or EGF(10 ng/ml) and Orlistat (100 µM) combination for 72 h. Significance in differences in cellular viability indices, in which vehicle acted as control, was determined by t test.

B. Western blot images showing phosphor-EGFR (Y1068), total EGFR, SREBP1 and FASN expression after vehicle, EGF (10 ng/ml) alone or EGF(10 ng/ml) and Orlistat (100 µM) combination treatments in NL20 cells for 72 h. ACTB was selected as a housekeeping gene control.

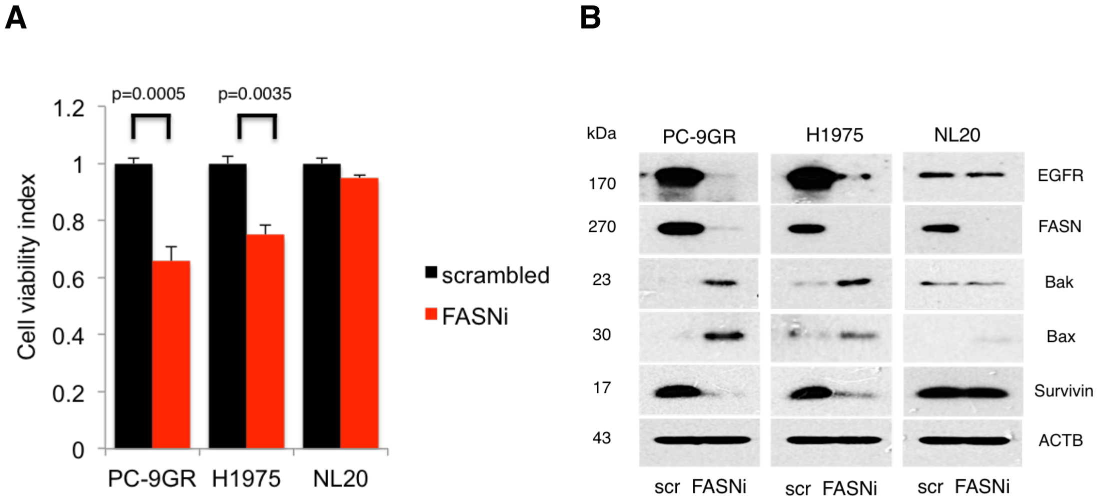

Appendix Fig S14. FASN knockdown by siRNAs in TKI resistant PC-9GR and H1975 cells.

A. Cell viability assays on the effect of FASN silencing (10 nM of FASN siRNAs) for 72 h) in NSCLC and NL20 cells. Significance in differences in cell viability indices, in which scrambled acted as control, was determined by t test.

B. Western blot analysis showing the effect of FASN silencing on EGFR, FASN, Bak, Bax and survivin expression. ACTB was selected as a housekeeping gene control.

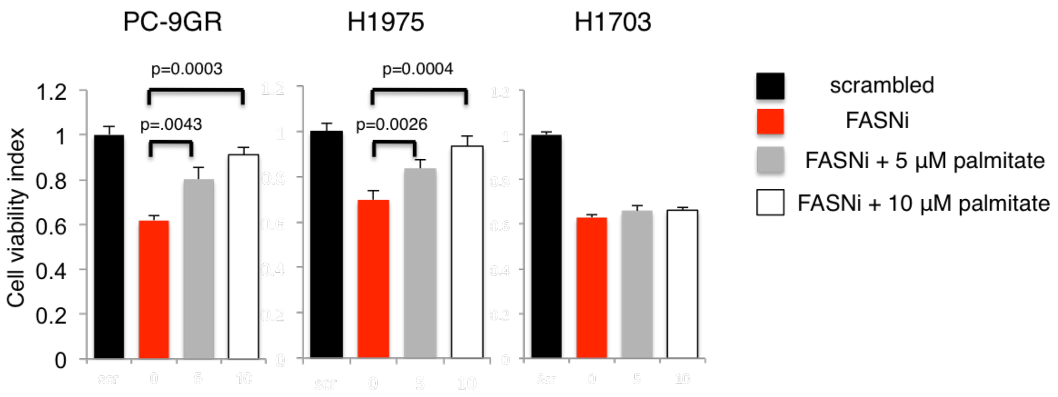

Appendix Fig S15. Cell viability assay showing the effects of exogenous palmitate supplementation to FASNi-treated PC-9GR, H1975 and H1703 NSCLC cells. Significance in differences in cell viability indices, in which scrambled acted as control, was determined by t test.

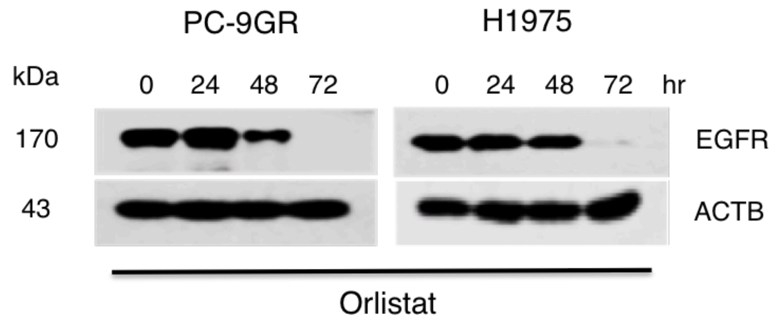

Appendix Fig S16. Western blot data showing EGFR expression after Orlistat (100 μM) treatment, at 24, 48 and 72 h, in PC-9GR and H1975 NSCLC cells. ACTB was selected as a housekeeping gene control.

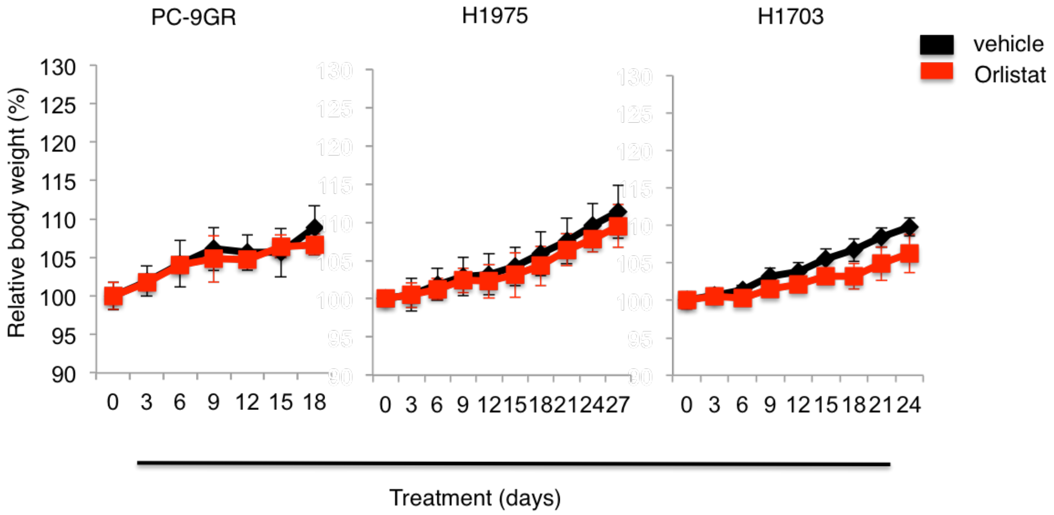

Appendix Fig S17. Comparison of body weight between vehicle- and Orlistat-treated mice carrying xenografts of PC-9GR and H1975 NSCLC cells. Orlistat – 240 mg/kg.

Sample	Luminescence Ratio B/A	Conclusion
Negative control	0.195	negative
Positive control	92.42	positive
PC-9P	0.297	negative
PC-9GR	0.422	negative
H1975	0.223	negative
H1703	0.169	negative
NL20	0.141	negative
HCC4006P	0.164	negative
HCC4006GR	0.212	negative
H820	0.111	negative
H1650	0.209	negative

Intepretation of:		
Ratio of B/A	<1	negative
	1-1.2	borderline
	>1.2	positive

Appendix Fig S18. Results and Interpretation of MycoAlertT assay results on lung cells used in this study.

Centre for Translational Research and Diagnostics
Centre for Translational Medicine Level 11
National University of Singapore
14 Medical Drive
Singapore 117599
Office: +65 65168055, Lab: +65 65165043, Fax: +65 68749664

Cell ID Report

Prepared by:	Sarah Low Hong Hui
Date Prepared:	16 October 2013

1 Sample Information

Run Loading Number	18
Sample ID	PC-9
Additional ID Comments	-
Pellet/DNA	-
Estimated Cells in Pellet	-
Received by	-
Date Pellet Received	-
Date DNA Received	-
DNA vol. (µl)	-
DNA conc. (ng/µl)	-
DNA quality (A260/280)	-

DNA Extraction: DNeasy Blood and Tissue Kit (Qiagen, Hilden, Germany)
DNA Analysis: Nanodrop ND1000 (Wilmington, DE)

2 Run Information

Volume DNA Used (µl)	-
Amount DNA Used (ng)	-
Run Date	9th October 2013
Run File Name	Kong_20131009

Cell ID Analysis: Cell ID system (Promega, Madison, WI)
Capillary Electrophoresis: ABI 3130xl Genetic Analyser (Life Technologies, Foster City, CA)

Centre for Translational Research and Diagnostics
Centre for Translational Medicine Level 11
National University of Singapore
14 Medical Drive
Singapore 117599
Office: +65 65168055, Lab: +65 65165043, Fax: +65 68749664

Cell ID Report

Prepared by:	Sarah Low Hong Hui
Date Prepared:	16 October 2013

1 Sample Information

Run Loading Number	10
Sample ID	H1703
Additional ID Comments	-
Pellet/DNA	-
Estimated Cells in Pellet	-
Received by	-
Date Pellet Received	-
Date DNA Received	-
DNA vol. (µl)	-
DNA conc. (ng/µl)	-
DNA quality (A260/280)	-

DNA Extraction: DNeasy Blood and Tissue Kit (Qiagen, Hilden, Germany)
DNA Analysis: Nanodrop ND1000 (Wilmington, DE)

2 Run Information

Volume DNA Used (µl)	-
Amount DNA Used (ng)	-
Run Date	9th October 2013
Run File Name	Kong_20131009

Cell ID Analysis: Cell ID system (Promega, Madison, WI)
Capillary Electrophoresis: ABI 3130xl Genetic Analyser (Life Technologies, Foster City, CA)

Centre for Translational Research and Diagnostics
Centre for Translational Medicine Level 11
National University of Singapore
14 Medical Drive
Singapore 117599
Office: +65 65168055, Lab: +65 65165043, Fax: +65 68749664

Cell ID Report

Prepared by:	Sarah Low Hong Hui
Date Prepared:	16 October 2013

1 Sample Information

Run Loading Number	11
Sample ID	H1975
Additional ID Comments	-
Pellet/DNA	-
Estimated Cells in Pellet	-
Received by	-
Date Pellet Received	-
Date DNA Received	-
DNA vol. (µl)	-
DNA conc. (ng/µl)	-
DNA quality (A260/280)	-

DNA Extraction: DNeasy Blood and Tissue Kit (Qiagen, Hilden, Germany)
DNA Analysis: Nanodrop ND1000 (Wilmington, DE)

2 Run Information

Volume DNA Used (µl)	-
Amount DNA Used (ng)	-
Run Date	9th October 2013
Run File Name	Kong_20131009

Cell ID Analysis: Cell ID system (Promega, Madison, WI)
Capillary Electrophoresis: ABI 3130xl Genetic Analyser (Life Technologies, Foster City, CA)

Cell Line Authentication Service
STR Profile Report

Sample Submitted By: Teo Jun Ting

Email Address: teo.jun.ting83@gmail.com

Order ID: AITB-CLA 001

Cell Line Designation: CRL-2871

Date Sample Received: 4th Oct 2017 **Report Date:** 13th Oct 2017

Methodology: Ten short tandem repeat (STR) loci plus the gender determining locus, Amelogenin, were amplified using the commercially available GenePrint ® 10 System from Promega. The cell line sample was processed using the ABI Prism® 3730xl Genetic Analyzer. Data were analyzed using GeneMapper® 4.1 software (Applied Biosystems). Appropriate positive and negative controls were run and confirmed for each sample submitted. Known reference profiling against the ATCC STR database with the STR profile for your human cell line.

Data Interpretation: Cell lines were authenticated using Short Tandem Repeat (STR) analysis as described in 2012 in ANSI Standard (ASN-0002) by the ATCC Standards Development Organization (SDO) and in Capes-Davis et al., Match criteria for human cell line authentication: Where do we draw the line? Int. J. Cancer. 2012 Nov 8. doi: 0.1002/ijc.27931

**Test Results for Submitted Sample**					**ATCC Reference Database Profile**			
**Loci**	**Query Profile: HCC4006**				**Database Profile: CRL-2871**			
**TH01**	7				7			
**D21S11**	31							
**D5S818**	12				12			
**D13S317**	11	12			11	12		
**D7S820**	9	12			9	12		
**D16S539**	11	12			11	12		
**CSF1PO**	10				10			
**Amelogenin**	X				X			
**Vwa**	16	17			16	17		
**TPOX**	8	9			8	9		
Number of shared alleles between query sample and database profile:								14
Total number of alleles in the database profile:								14
Percent match between in the submitted sample and the database profile:								100
The alleles match algorithm compares the 8 core loci plus amelogenin only, even though alleles from all loci will be reported when available.								
**NOTE:** Loci highlighted in grey (8 core STR loci plus Amelogenin) can be made public to verify cell identity. In order to protect the identity of the donor, please do not publish the allele calls from the STR loci tested. Electropherograms showing raw data are attached.								

Explanation of Test Results
Cell lines with ≥ 80% match are considered to be related; i.e., derived from a common ancestry.
Cell lines with between a 55% to 80% match require further profiling for authentication of relatedness.

☐ The submitted sample profile is human, but not a match for any profile in the ATCC STR database
√ The submitted sample is an exact match for the following ATCC human cell line(s) in the ATCC STR database (8 core loci plus Amelogenin): CRL-2871
☐ The submitted sample is similar to the following ATCC human cell line(s):

**Performed By Lab Technician:**	Jennifer Ng 13/10/2017
**Reviewed By:**	Sathya 13/10/2017

Cell Line Authentication Service
STR Profile Report

Sample Submitted By: Teo Jun Ting

Email Address: teo.jun.ting83@gmail.com

Order ID: AITB-CLA 001

Cell Line Designation: CRL-2503

Date Sample Received: 4th Oct 2017 **Report Date:** 13th Oct 2017

Methodology: Ten short tandem repeat (STR) loci plus the gender determining locus, Amelogenin, were amplified using the commercially available GenePrint ® 10 System from Promega. The cell line sample was processed using the ABI Prism® 3730xl Genetic Analyzer. Data were analyzed using GeneMapper® 4.1 software (Applied Biosystems). Appropriate positive and negative controls were run and confirmed for each sample submitted. Known reference profiling against the ATCC STR database with the STR profile for your human cell line.

Data Interpretation: Cell lines were authenticated using Short Tandem Repeat (STR) analysis as described in 2012 in ANSI Standard (ASN-0002) by the ATCC Standards Development Organization (SDO) and in Capes-Davis et al., Match criteria for human cell line authentication: Where do we draw the line? Int. J. Cancer. 2012 Nov 8. doi: 0.1002/ijc.27931

**Test Results for Submitted Sample**					**ATCC Reference Database Profile**			
**Loci**	**Query Profile: NL-20**				**Database Profile: CRL-2503**			
**TH01**	6	7			6	7		
**D21S11**	29	31.2						
**D5S818**	12				12			
**D13S317**	11	12			11	12		
**D7S820**	12				12			
**D16S539**	12				12			
**CSF1PO**	11				11			
**Amelogenin**	X				X			
**Vwa**	14	17			14	17		
**TPOX**	9	11			9	11		
Number of shared alleles between query sample and database profile:								13
Total number of alleles in the database profile:								13
Percent match between in the submitted sample and the database profile:								100
The alleles match algorithm compares the 8 core loci plus amelogenin only, even though alleles from all loci will be reported when available.								
**NOTE:** Loci highlighted in grey (8 core STR loci plus Amelogenin) can be made public to verify cell identity. In order to protect the identity of the donor, please do not publish the allele calls from the STR loci tested. Electropherograms showing raw data are attached.								

Explanation of Test Results

Cell lines with ≥ 80% match are considered to be related; i.e., derived from a common ancestry.

Cell lines with between a 55% to 80% match require further profiling for authentication of relatedness.

☐ The submitted sample profile is human, but not a match for any profile in the ATCC STR database

√ The submitted sample is an exact match for the following ATCC human cell line(s) in the ATCC STR database (8 core loci plus Amelogenin): CRL-2503

☐ The submitted sample is similar to the following ATCC human cell line(s):

**Performed By Lab Technician:**	Jennifer Ng 13/10/2017
**Reviewed By:**	Sathya 13/10/2017

Cell Line Authentication Service
STR Profile Report

Sample Submitted By: Teo Jun Ting

Email Address: teo.jun.ting83@gmail.com

Order ID: AITB-CLA 001

Cell Line Designation: HTB-181

Date Sample Received: 4th Oct 2017 **Report Date:** 13th Oct 2017

Methodology: Ten short tandem repeat (STR) loci plus the gender determining locus, Amelogenin, were amplified using the commercially available GenePrint ® 10 System from Promega. The cell line sample was processed using the ABI Prism® 3730xl Genetic Analyzer. Data were analyzed using GeneMapper® 4.1 software (Applied Biosystems). Appropriate positive and negative controls were run and confirmed for each sample submitted. Known reference profiling against the ATCC STR database with the STR profile for your human cell line.

Data Interpretation: Cell lines were authenticated using Short Tandem Repeat (STR) analysis as described in 2012 in ANSI Standard (ASN-0002) by the ATCC Standards Development Organization (SDO) and in Capes-Davis et al., Match criteria for human cell line authentication: Where do we draw the line? Int. J. Cancer. 2012 Nov 8. doi: 0.1002/ijc.27931

Test Results for Submitted Sample					ATCC Reference Database Profile			
**Loci**	**Query Profile: NCI-H820**				**Database Profile: HTB-181**			
**TH01**	8				8			
**D21S11**	30							
**D5S818**	9	11			9	11		
**D13S317**	12				12			
**D7S820**	10	13			10	13		
**D16S539**	9	11			9	11		
**CSF1PO**	11	12			11	12		
**Amelogenin**	X	Y			X	Y		
**Vwa**	18				18			
**TPOX**	12				12			
Number of shared alleles between query sample and database profile:								14
Total number of alleles in the database profile:								14
Percent match between in the submitted sample and the database profile:								100
The alleles match algorithm compares the 8 core loci plus amelogenin only, even though alleles from all loci will be reported when available.								
**NOTE:** Loci highlighted in grey (8 core STR loci plus Amelogenin) can be made public to verify cell identity. In order to protect the identity of the donor, please do not publish the allele calls from the STR loci tested. Electropherograms showing raw data are attached.								

Explanation of Test Results
Cell lines with ≥ 80% match are considered to be related; i.e., derived from a common ancestry.
Cell lines with between a 55% to 80% match require further profiling for authentication of relatedness.

☐ The submitted sample profile is human, but not a match for any profile in the ATCC STR database
√ The submitted sample is an exact match for the following ATCC human cell line(s) in the ATCC STR database (8 core loci plus Amelogenin): HTB-181
☐ The submitted sample is similar to the following ATCC human cell line(s):

**Performed By Lab Technician:**	Jennifer Ng 13/10/2017
**Reviewed By:**	Sathya 13/10/2017

Centre for Translational Research and Diagnostics
Centre for Translational Medicine Level 11
National University of Singapore
14 Medical Drive
Singapore 117599
Office: +65 65168055, Lab: +65 65165043, Fax: +65 68749664

Cell ID Report

Prepared by:	Sarah Low Hong Hui
Date Prepared:	16 October 2013

1 Sample Information

Run Loading Number	8
Sample ID	H1650
Additional ID Comments	-
Pellet/DNA	-
Estimated Cells in Pellet	-
Received by	-
Date Pellet Received	-
Date DNA Received	-
DNA vol. (µl)	-
DNA conc. (ng/µl)	-
DNA quality (A260/280)	-

DNA Extraction: DNeasy Blood and Tissue Kit (Qiagen, Hilden, Germany)
DNA Analysis: Nanodrop ND1000 (Wilmington, DE)

2 Run Information

Volume DNA Used (µl)	-
Amount DNA Used (ng)	-
Run Date	9th October 2013
Run File Name	Kong_20131009

Cell ID Analysis: Cell ID system (Promega, Madison, WI)
Capillary Electrophoresis: ABI 3130xl Genetic Analyser (Life Technologies, Foster City, CA)

Appendix Fig S19. Cell line authentication of cell lines used in this study.

Appendix Table S1

Identification of the top 10 differentially regulated fatty acid metabolic genes in acquired resistant PC-9GR cells compared to parental PC-9P cells). Genes listed are Log2 > 0.6 values (Fold-change of >1.5) and p-value < 0.05.

**Fatty acid metabolic process**		
Rank #	Gene Name	Log2
1	FASN	0.966
2	NR2F2	0.782
3	SREBF1	0.722
4	FDXR	0.702
5	ALDH5A1	0.657
6	THRSP	0.648
7	ST6GALNAC4	0.629
8	PCYT2	0.623
9	PIGV	0.588
10	IMPA1	0.585

Appendix Table S2. Q-PCR validation of the top 10 fatty acid metabolic genes from microarray studies. ACTB housekeeping gene was used for normalization.

**Rank #**	**Gene name**	**Microarray Fold-change**	**Q-PCR Fold-change (SEM)**
1	FASN	1.95	15.18 (0.021)
2	NR2F2	1.72	2.33 (0.08)
3	SREBF1	1.65	11.04 (0.228)
4	FDXR	1.63	1.53 (0.07)
5	ALDH5A1	1.58	3.84 (0.126)
6	THRSP	1.57	3.01 (0.05)
7	ST6GALNAC4	1.55	2.55 (0.09)
8	PCYT2	1.54	4.66 (0.08)
9	PIGV	1.5	1.38 (0.03)
10	IMPA1	1.5	1.82 (0.03)

Appendix Table S3. EGFR mutation status in lung cells adopted in this study.

Lung cell lines			
**Cell line name**	**Type**	**EGFR status**	**Gefitinib resistance**
PC-9P	NSCLC	delE746-A750	No
PC-9GR	NSCLC	delE746-A750	Yes
H1975	NSCLC	T790M/L858R	Yes
H1703	NSCLC	Wild-type	Yes
NL20	Normal lung	Wild-type	Yes
HCC4006P	NSCLC	delL747-A749/A750P	No
HCC4006GR	NSCLC	delL747-A749/A750P	Yes
H1650	NSCLC	delE746-A750	Yes
H820	NSCLC	delE746-A749/T790M	Yes

Appendix Table S4. List of primer sequences used for quantitative PCR in this study.

Gene		Sequence (5' - '3)	PrimerBank ID	Amplicon size (bp)
FASN	F	AAGGACCTGTCTAGGTTTGATGC	41872630c1	106
	R	TGGCTTCATAGGTGACTTCCA		
NR2F2	F	TCATGGGTATCGAGAACATTTGC	223555950c1	151
	R	TTCAACACAAACAGCTCGCTC		
SREBF1	F	GCCCCTGTAACGACCACTG	256665250c2	84
	R	CAGCGAGTCTGCCTTGATG		
FDXR	F	CTGAGGCAGAGTCGAGTGAAG	111118982c1	85
	R	CCCGAAGCTCCTTAATGGTGA		
ALDH5A1	F	AGGGGAGGCAATTTGTACTGA	25777720c3	85
	R	GTGGTGCAACAGGATCTTTCC		
THRSP	F	CAGGTGCTAACCAAGCGTTAC	318037251c1	108
	R	CAGAAGGCTGGGGATCATCA		
ST6GALNAC4	F	TGTGAGGAGATCGTGGTCTATG	88999589c2	85
	R	CAAAGTAGTGGTAAGGCACTGAG		
PCYT2	F	TGTCCACCACAGACCTCGT	374253759c1	89
	R	ATACTCCCGGTACTCAGAGGA		
PIGV	F	ACACCCAATTCTGTCTGCCAG	322309841c3	78
	R	GGTAGCCCTTGTCTACAGCTAAC		
IMPA1	F	TAACTCTAGCAAGACAAGCTGGA	221625506c1	115
	R	TCAACTTTTTGGTCCGTAGCAG		
ACTB	F	CATGTACGTTGCTATCCAGGC	4501885a1	250
	R	CTCCTTAATGTCACGCACGAT		
tGFP	F	AGGACAGCGTGATCTTCACC		92
	R	GCTGCCATCCAGATCGTTAT		
