# Supplementary material for: Fatty acid synthase mediates EGFR palmitoylation in EGFR mutated non‐small cell lung cancer
Source: EMBO Mol Med. 2018 Feb 15;10(3):e8313. doi: 10.15252/emmm.201708313 (PMC5840543; doi:10.15252/emmm.201708313)
Supplement: Supplementary file 3 — Source Data for Figure 2D [file EMMM-10-e8313-s002.pptx]

## Slide 1
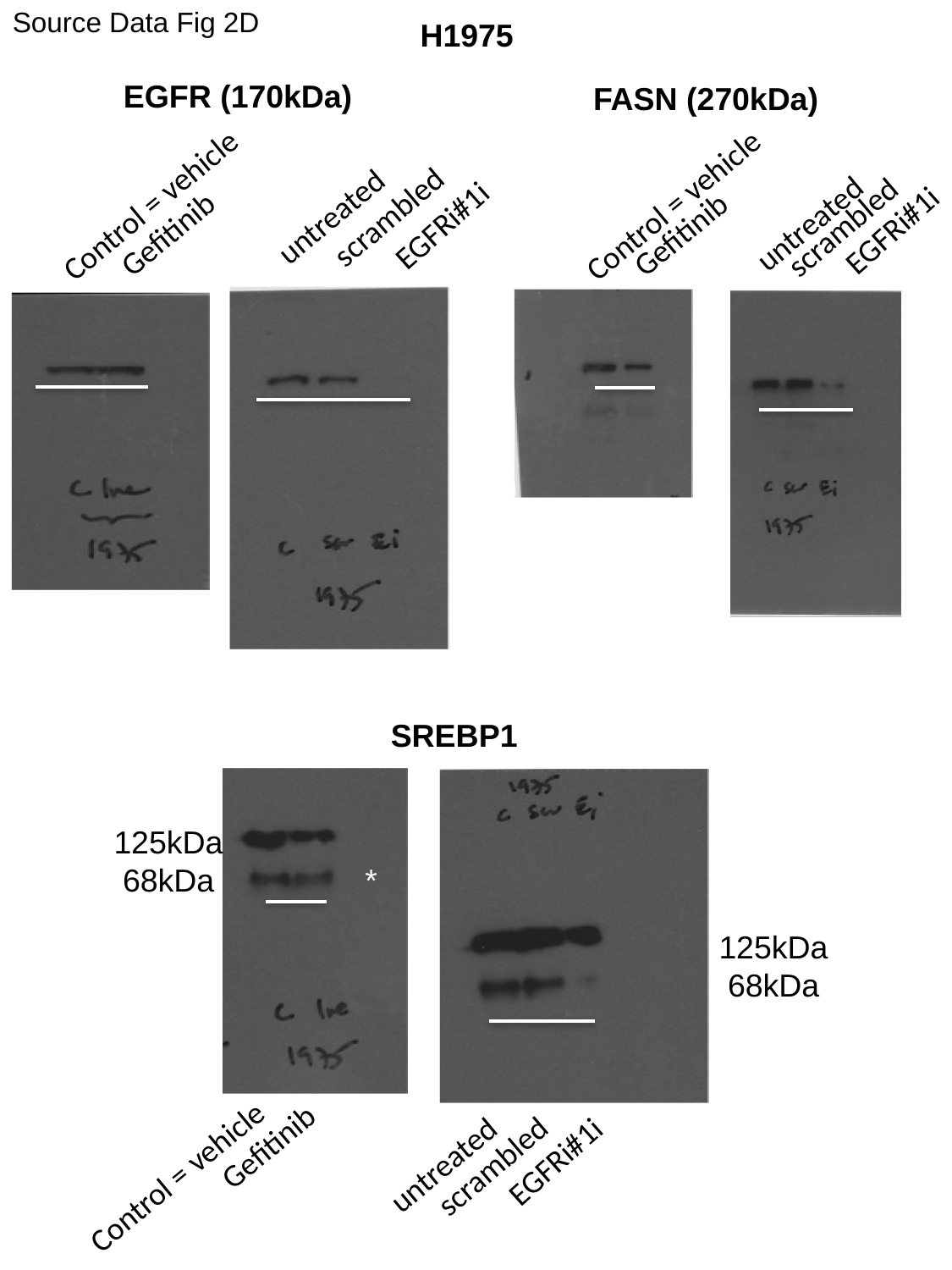

Source Data Fig 2D
H1975
EGFR (170kDa)
Control = vehicle
untreated
scrambled
EGFRi#1i
Gefitinib
FASN (270kDa)
*
Control = vehicle
untreated
scrambled
EGFRi#1i
Gefitinib
SREBP1
*
125kDa
68kDa
125kDa
68kDa
Gefitinib
EGFRi#1i
untreated
scrambled
Control = vehicle
