# Supplementary material for: Fatty acid synthase mediates EGFR palmitoylation in EGFR mutated non‐small cell lung cancer
Source: EMBO Mol Med. 2018 Feb 15;10(3):e8313. doi: 10.15252/emmm.201708313 (PMC5840543; doi:10.15252/emmm.201708313)
Supplement: Supplementary file 4 — Source Data for Figure 3C [file EMMM-10-e8313-s003.pptx]

## Slide 1
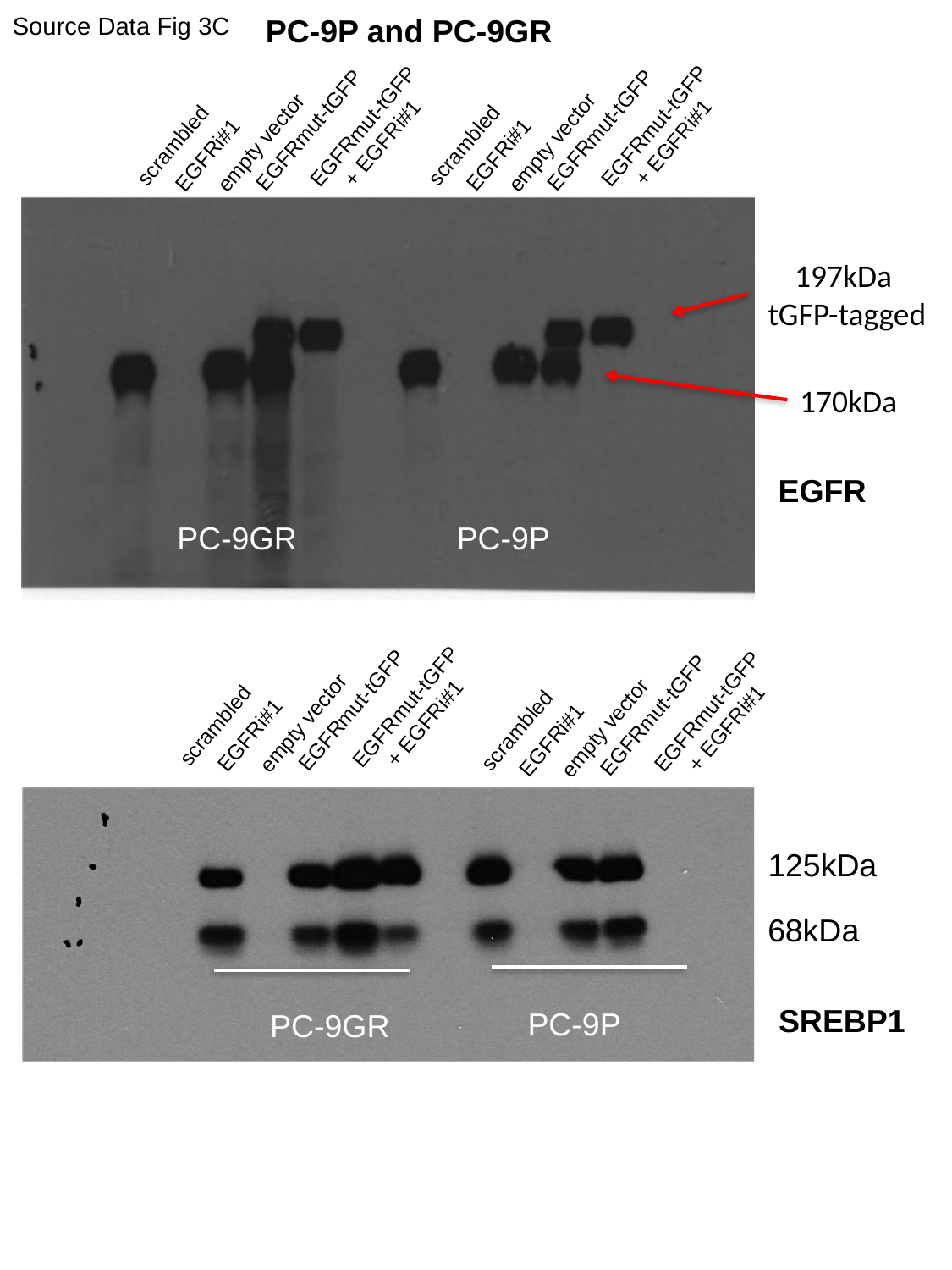

PC-9P and PC-9GR
Source Data Fig 3C
EGFRmut-tGFP
+ EGFRi#1
EGFRmut-tGFP
empty vector
scrambled
EGFRi#1
EGFRmut-tGFP
+ EGFRi#1
EGFRmut-tGFP
empty vector
scrambled
EGFRi#1
PC-9GR
PC-9P
197kDa
 tGFP-tagged
170kDa
EGFR
EGFRmut-tGFP
+ EGFRi#1
EGFRmut-tGFP
empty vector
scrambled
EGFRi#1
EGFRmut-tGFP
+ EGFRi#1
EGFRmut-tGFP
empty vector
scrambled
EGFRi#1
PC-9P
PC-9GR
125kDa
68kDa
SREBP1
