# Supplementary material for: Fatty acid synthase mediates EGFR palmitoylation in EGFR mutated non‐small cell lung cancer
Source: EMBO Mol Med. 2018 Feb 15;10(3):e8313. doi: 10.15252/emmm.201708313 (PMC5840543; doi:10.15252/emmm.201708313)
Supplement: Supplementary file 5 — Source Data for Figure 4C [file EMMM-10-e8313-s004.pptx]

## Slide 1
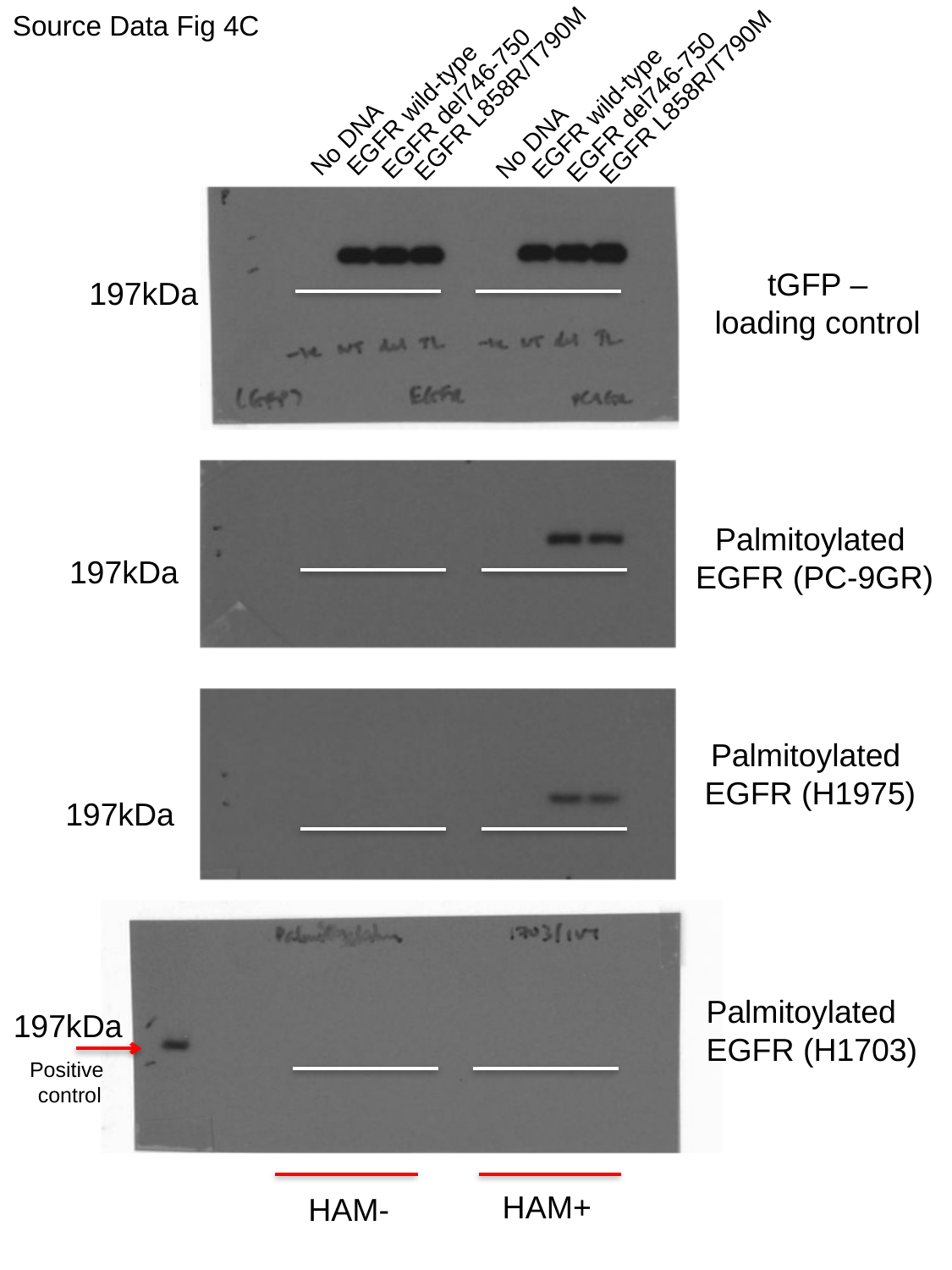

EGFR L858R/T790M
EGFR del746-750
EGFR wild-type
No DNA
EGFR L858R/T790M
EGFR del746-750
EGFR wild-type
No DNA
tGFP –
loading control
197kDa
Palmitoylated
EGFR (PC-9GR)
197kDa
Palmitoylated
 EGFR (H1975)
197kDa
Palmitoylated
EGFR (H1703)
197kDa
Positive
control
HAM+
HAM-
Source Data Fig 4C
