# Supplementary material for: Fatty acid synthase mediates EGFR palmitoylation in EGFR mutated non‐small cell lung cancer
Source: EMBO Mol Med. 2018 Feb 15;10(3):e8313. doi: 10.15252/emmm.201708313 (PMC5840543; doi:10.15252/emmm.201708313)
Supplement: Supplementary file 6 — Source Data for Figure 5E [file EMMM-10-e8313-s005.pptx]

## Slide 1
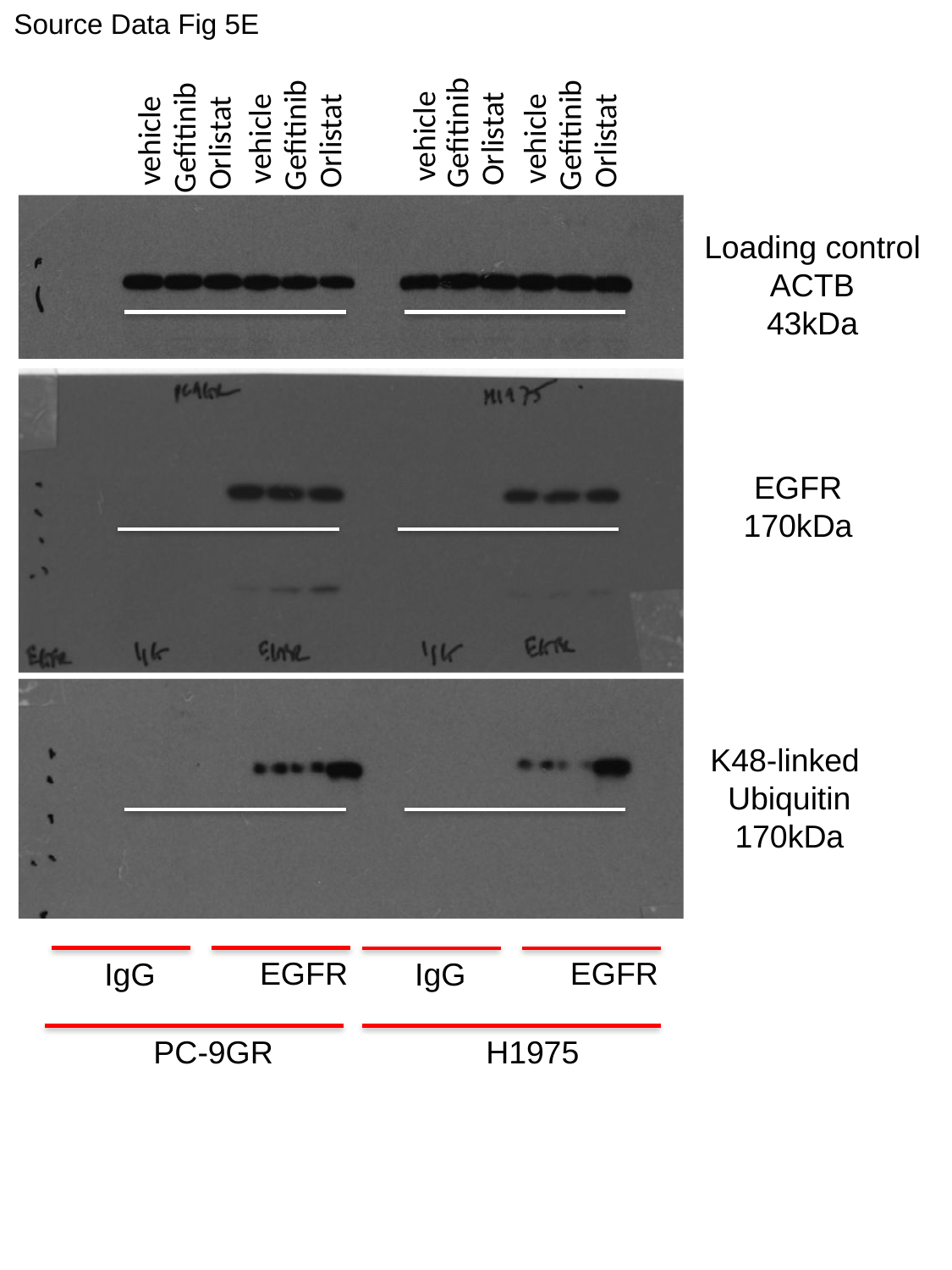

Source Data Fig 5E
Gefitinib
vehicle
Orlistat
Gefitinib
vehicle
Orlistat
Gefitinib
vehicle
Orlistat
Gefitinib
vehicle
Orlistat
Loading control
ACTB
43kDa
EGFR
170kDa
K48-linked
Ubiquitin
170kDa
PC-9GR
H1975
EGFR
EGFR
IgG
IgG
PC-9GR
H1975
