# Supplementary figures and images for: Fatty acid synthase mediates EGFR palmitoylation in EGFR mutated non‐small cell lung cancer
Source: EMBO Mol Med. 2018 Feb 15;10(3):e8313. doi: 10.15252/emmm.201708313 (PMC5840543; doi:10.15252/emmm.201708313)

## Slide 1
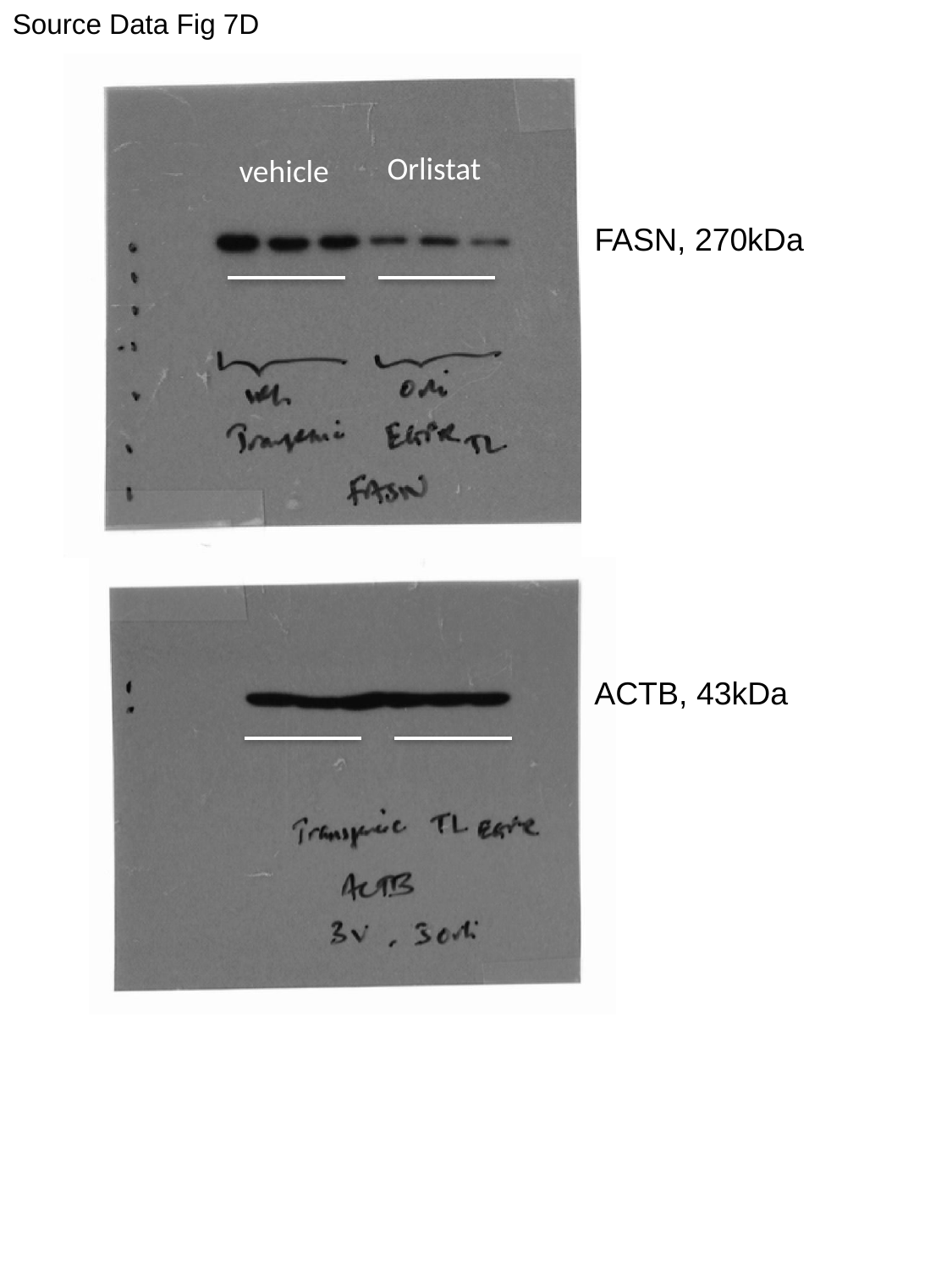

Source Data Fig 7D
Orlistat
vehicle
FASN, 270kDa
ACTB, 43kDa

Supplement: Supplementary file 7 — Source Data for Figure 7D [file EMMM-10-e8313-s006.pptx]
